# Regulation of Src Family Kinases in Human Cancers

**DOI:** 10.1155/2011/865819

**Published:** 2011-04-04

**Authors:** Banibrata Sen, Faye M. Johnson

**Affiliations:** ^1^Department of Thoracic/Head & Neck Medical Oncology, The University of Texas MD Anderson Cancer Center, Houston, TX 77030, USA; ^2^The University of Texas Graduate School of Biomedical Sciences at Houston, Houston, TX 77030, USA

## Abstract

The nonreceptor protein tyrosine kinase Src plays a crucial role in the signal transduction pathways involved in cell division, motility, adhesion, and survival in both normal and cancer cells. Although the Src family kinases (SFKs) are activated in various types of cancers, the exact mechanisms through which they contribute to the progression of individual tumors remain to be defined. The activation of Src in human cancers may occur through a variety of mechanisms that include domain interaction and structural remodeling in response to various activators or upstream kinases and phosphatastes. Because of Src's prominent roles in invasion and tumor progression, epithelial-to-mesenchymal transition, angiogenesis, and the development of metastasis, Src is a promising target for cancer therapy. Several small molecule inhibitors of Src are currently being investigated in clinical trials. In this article, we will summarize the mechanisms regulating Src kinase activity in normal and cancer cells and discuss the status of Src inhibitor development against various types of cancers.

## 1. Introduction

Francis Peyton Rous was awarded the Nobel prize in 1966 for his groundbreaking discovery that a virus could cause cancer [1]. In 1911, he was able to purify a substance from chickens that was later shown to be a sarcoma-causing virus (Rous sarcoma virus). The responsible oncogene was called *v-Src* [2, 3]. In 1976, J. M. Bishop and H. E. Varmus discovered a related gene in chickens, which showed a striking resemblance to *v-Src*. This normal cellular counterpart, cellular *Src* (known as *c-Src* or *Src*), was the first proto-oncogene to be identified, and its discovery led to the Nobel prize for medicine in 1989 [2]. Src was also the first gene product discovered to have intrinsic protein tyrosine kinase activity [4–6].

Src belongs to a family of 11 nonreceptor tyrosine kinases known as the Src family kinases (SFKs); the other ten are Fyn, Yes, Blk, Yrk, Frk (also known as Rak), Fgr, Hck, Lck, Srm, and Lyn. The human genome contains a Yes pseudogene (YESps), and Src, Yes, YESps, and Fyn are ubiquitously expressed in a variety of tissues [7, 8]. Srm is found in keratinocytes, whereas Blk, Fgr, Hck, Lck, and Lyn are found primarily in hematopoietic cells. Frk occurs chiefly in bladder, breast, brain, colon, and lymphoid cells. Like all members of the Src kinase family, the Frk kinase possesses an SH domain as well as conserved autoregulatory tyrosine residues in its catalytic domain [9, 10]. However, Frk differs significantly from the other Src family members in many structural features, including the presence of a putative bipartite nuclear localization signal and the lack of a consensus myristoylation motif [10, 11]. In fact, Frk has been shown to be a nuclear protein with growth-inhibitory effects when ectopically expressed in breast cancer cells [12]. Blk occurs chiefly in colon, prostate, and small intestine cells; however, it was initially isolated from a breast cancer cell line [13]. 

In this review, we will discuss the structure of SFKs, the regulation of their kinase activity, the involvement of SFKs in the development of cancer, and recent therapeutic advancements in targeting SFKs.

## 2. Structure of the Src Family Kinases

The ability of the avian viral oncoproteins v-Src and v-Yes to induce fibroblast transformation suggests that their cellular counterparts, Src and c-Yes, have the potential to contribute to human carcinogenesis. v-Src and v-Yes are encoded by avian retroviruses and are capable of inducing sarcomas in chickens and of transforming chicken embryo fibroblast cells in culture [14, 15]. To understand how these proteins are able to induce cell transformation, it is important to understand the functional domain architecture shared by all SFKs and the role of these domains in both regulating tyrosine kinase activity and recruiting additional proteins into signaling complexes. These aspects of SFK behavior have also been reviewed extensively elsewhere [8, 16]. 

**Figure 1:**
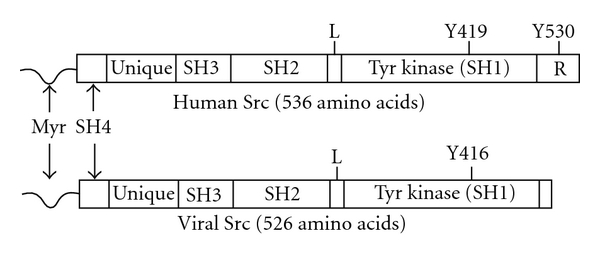
Schematic of the structural domain of human Src (Upper) and v-Src (Lower). The Src molecule is composed of an N-terminal myristoylation sequence (Myr) attached to the SH4 domain, a unique region followed by SH3 and SH2 domains, a linker region (L), a kinase domain (SH1 domain) that contains Tyr419, and a C-terminal regulatory domain (R) that contains Tyr530. v-Src protein differs from Src in a number of ways, with one major difference being the lack of a regulatory domain (R) at the C-terminal sequence.

Src is a 60-KDa protein composed of several functional domains [13, 17, 18]. Src contains a 14-carbon myristic acid moiety attached to an SH4 domain, a unique domain, an SH3 domain followed by an SH2 domain, an SH2-kinase linker, a protein tyrosine kinase domain (also known as an SH1 domain), and a C-terminal regulatory segment [19] (Figure 1). During cotranslational modification, the N-terminal methionine is removed and the resulting N-terminal glycine is myristoylated by myristoyl-coA. Myristoylation facilitates attachment to the inner surface of the cell membrane [19]. N-myristoylation is required for Src membrane association and its ability to transform cells [13, 20]. The differential state of palmitoylation at the SH4 domain of SFKs regulates subcellular trafficking of different SFKs in intact cell. All SFKs are cotranslationally myristoylated at Cly2 with the exception of Src and Blk, which are post translationally palmitoylated at Cys3, Cys5 or Cys6 [21]. Fatty acylation of SFKs has been shown to regulate their interaction with the cell membrane and their subcellular distribution [22, 23]. The poorly conserved unique domain is believed to provide unique functions and specificity to each SFK member. 

**Figure 2:**
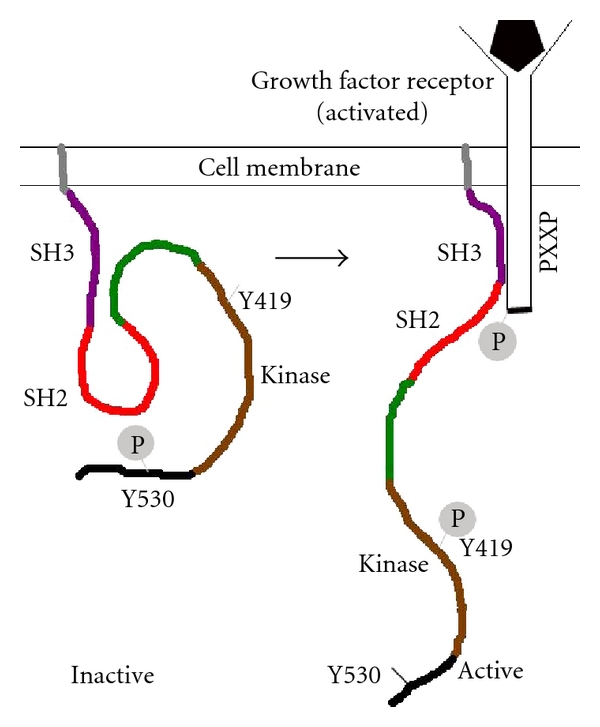
Cartoon representation of Src kinase regulation by differential phosphorylation at kinase domain as well as C-terminal regulatory domain.

 The SH3 domain, composed of ~60 amino acid residues, is able to bind proline-rich sequences facilitating SFK-substrate or intramolecular interactions [19, 24]. The SH2 domain is composed of ~100 amino acids that can bind to phosphorylated tyrosine residues on either its own C-terminal regulatory domain or those of other proteins. Songyang and Cantley analyzed the binding of a library of phosphopeptides to the SH2 domain to define the preferred docking sequence [25]. The SH2 domain of each SFK member has distinct peptide preferences towards its binding partners [26]. The linker domain is involved in intramolecular binding with the SH3 domain. The catalytic domain is composed of two subdomains separated by a catalytic cleft, in which the adenosine-5′-triphosphate (ATP) and substrate-binding sites reside and phosphate transfer occurs. The cleft forms an activation loop that contains Tyrosine 419 (Tyr419; human Src) which is the positive regulatory site responsible for maximizing kinase activity [19]. Phosphorylation at the C-terminal end Tyr530 (human Src), which is a negative regulatory residue, leads to binding of this region to the SH2 domain; thus a “closed” or inactive conformation is attained, which is inaccessible to external ligands. In the closed conformation, the activation loop attains a compact structure, which fills the catalytic cleft and masks Tyr419 residues, thus preventing Tyr419 autophosphorylation and subsequent activation (Figure 2).

## 3. Src Activation in Cancer

Src actions on mammalian cells are pleiotropic and include effects on cell morphology, adhesion, migration, invasion, proliferation, differentiation, and survival. Src kinase activation is common in various types of cancers although activating mutations and genomic amplifications are very rare. Thus, Src activation is usually accomplished by structural alteration mediated by upstream kinases or phosphatases. There are several ways SFKs activities are regulated, which include interactions that influence its intramolecular interactions and localizations. The net phosphorylation status of Src at its regulatory residues determines the activation status of Src, which is dependent upon a balance between phosphatase and kinase enzymes.

### 3.1. Regulation through the C-Terminal Negative Regulatory Domain

There are several ways Src kinase activity can be regulated, and any one of these might contribute to its activation in cancer cells. These include the phosphorylation of Tyr530, deletion or mutation of the C-terminal regulatory region, displacement of the SH3- and SH2 domain mediated by intramolecular interactions with higher affinity ligands, and phosphorylation of Tyr419. Independent biochemical and X-ray crystallographic analyses have revealed that Src maintains its inactive condition by various internal interactions. The interactions between the SH2 domain and the C-terminal Tyr530, as well as interactions between the SH3 domain and the SH2-kinase linker, modulate SFK activity [27]. 

Phosphorylation of the C-terminal negative regulatory tyrosine (Tyr530, human Src) is one of the mechanisms for the regulation of SFK activity. Due to the loss of the C-terminal residues, the viral proteins v-Src and v-Yes, are no longer able to be regulated by intramolecular interaction and become constitutively active and transformation competent [14, 15]. Regulation through the phosphorylation of Tyr530 in Src is accomplished by several kinases and phosphatases. 

Two important protein tyrosine kinases in this process are Csk (c-Src kinase) and its homolog Csk-homologous kinase (Chk), which are both able to phosphorylate Tyr530 and to inactivate Src [28–30]. Reduced expression of Csk might play a role in the activation of Src in some cancers. In hepatocellular carcinoma, Csk levels are reduced compared to those in normal liver tissue and this reduced expression correlates with enhanced Src activity [31]. Evidence suggests that overexpression of Csk also appears to reduce tumor metastasis in colon cancer [32]. In addition to the reduced expression of Csk seen in cancer cells, other modes of regulating Csk are now being identified. Csk is structurally similar to Src, but its mode of regulation is distinct in that it lacks the regulatory tyrosine residue at the C-terminal end to control its activity [33]. 

 Another mechanism of the regulation of Csk is through the transmembrane adaptor protein Cbp (Csk-binding protein or protein associated with glycosphingolipid-enriched microdomains [PAG]), a lipid raft-associated binding partner of Csk. Following phosphorylation by Src, Cbp can bind to the SH2 domain of Csk, thus allowing its recruitment to the plasma membrane where active Src resides. This creates a negative regulatory loop in which Cbp mediates the cross-linking of active Src with its suppressor, Csk [34]. An independent study by Oneyama et al. showed that membrane-bound adaptor protein Cbp suppress the Src-mediated cell transformation and tumorigenesis by binding and sequestering Src within lipid rafts [35]. Interestingly, this Cbp-mediated Src suppression was Csk independent. They have shown that Csk−/− mouse embryonic fibroblast cells underwent malignant transformation in the presence of Src [36]. The authors first noted that the levels of endogenous Cbp messenger RNA and protein were reduced when activated Src was expressed. They then made the seminal observation that overexpression of exogenous Cbp reversed the oncogenic effect of Src. They found that Cbp did not have any effect on Src tyrosine kinase activity; instead, it altered Src localization. The SH2 domain of Src binds to tyrosine phosphorylated Cbp and moves to the raft region and becomes inaccessible to kinase action. The cytoplasmic domain of Cbp has two proline-rich SH3 binding motifs and ten tyrosine residues, nine of which are Src targets. Oneyama found that phosphorylated Cbp could recruit SH2 domain-containing proteins such as Csk, SFKs, and suppressor of cytokine signaling 1 (SOCS1) to lipid rafts [34]. This finding further complicated our understanding of lipid rafts. Previous evidence had suggested that lipid rafts acted as positive hubs for activated signaling molecules and their associated SFKs. In order to mediate signals, SFKs need to be localized to the raft region [37]. Moreover, two independent studies have shown that SFKs remain active and can drive cancer cell growth even when bound to lipid-raft associated Cbps [38, 39]. This conflict can be addressed by studying the differences in fatty acylation status, cell types, and extent of Cbp interaction with SFKs.

## 4. Regulation of Src Activity by Phosphatases

Several protein tyrosine phosphatases (PTPs) are able to dephosphorylate Src Tyr530 and are responsible for the regulation of its kinase activity, such as PTP*α*,  PTP*γ*, SHP-1 and -2, and PTP1B. PTP*α* is ubiquitously expressed and enriched in brain tissue [40–42] and is also able to dephosphorylate Tyr419, as evidenced by the lack of pSrcTyr419 in PTP*α*-overexpressing cells [40, 43]. Overexpression of PTP*α* also can dephosphorylate Src in A431 cell lines and cause enhancements in cell adhesion [44, 45]. A general question arises from these studies as to whether PTP*α* acts as an activator or repressor of Src molecules. Antisense studies of PTP*α* in 3T3-L1 adipocytes [46] and PTP*α*−/− murine studies [47, 48] show that Src kinase activity is linearly correlated with levels of PTP*α*  protein in cells. 

PTP*γ* was first identified from chicken brain tissue as a homolog of CD45 capable of dephosphorylating the SFK Lck [49]. It is expressed in the spleen and intestine and is able to dephosphorylate both Tyr530 and Tyr419 residues in Src. Chappel et al. have shown that PTP*γ* can modulate Src activity in osteoclast precursor cells treated with 1,25-dihydroxyvitamine D3; there was a dramatic increase in Src kinase activity without an increase in total protein levels. This change was accompanied by a decrease in phosphorylation at Tyr530 Interestingly both PTP*γ* mRNA and PTP*γ* protein levels were upregulated upon 1,25-dihydroxyvitamine D3 treatment suggesting the possibility that PTPg might be responsible for elevated Src kinase activity [50]. 

SHP1 is another member of the protein tyrosine phosphatase protein family that is also known as PTP-1c. It is a cytosolic two-SH2 domain containing PTP expressed in epithelial and hematopoetic cells [51]. Somani et al. have shown that SHP1 is responsible for the dephosphorylation and subsequent activation of Src, and it is much more specific for Src Tyr530 than Tyr419. This observation has been validated in transgenic mice that expressed the mutated loss of function form of SHP1, which has an increased level of Tyr530-phosphorylated Src [52]. 

SHP2 is a cytoplasmic SH2 domain containing PTP, which is also able to dephosphorylate Tyr530 [53]. SHP2 is very specific for the C-terminal regulatory tyrosine residue of Src. An independent study by Walter et al. demonstrated that SHP2 overexpression led to the activation of Src without significant changes in tyrosine phosphorylation at either residue (Tyr419 or Tyr530). In addition, the phosphatase-inactive mutant of SHP2 was also capable of Src activation. Further studies on the mechanism of Src activation by SHP2 revealed that the SH2 domain of SHP2 associates with Src by binding to the Src-SH3 domain and results in the allosteric activation of Src without involving Src dephosphorylation [54].

Another tyrosine phosphatase known as PTP-1B (also known as PTPN1) was first identified by Charbonneau et al. and first cloned and purified from human placenta [55–59]. Later Bjorge et al. demonstrated that PTP-1B was associated with Src activation in breast cancer cell lines [60]. PTP-1B is capable of both *in vitro* and *in vivo* activation of Src kinase activity as a result of its specificity towards tyrosine residues at the C-terminal tail. Human melanocyte [61] and several breast cancer cell lines [62] have elevated Src activity with concomitant hypophosphorylation of Tyr530. Biochemical analyses showed that these cells have elevated levels of PTP activity, which correlates with reduced phosphorylation on the C-terminal residue of Src and may have an important role in controlling Src kinase activity. The ability of PTP-1B to modulate Src activity has been demonstrated in mouse L-cell fibroblasts [63]. 

 Rare activating mutations in *Src* that are truncated at codon 531 have been reported in some cases of advanced colon cancer patients [64]. The *Src* 531 mutation resulted in the production of a stop at codon 531, one residue beyond the regulatory Tyr530. Due to the lack of a C-terminal regulatory region, phosphorylation of Tyr530 did not result in a closed conformation and the mutated Src remained constitutively active.

## 5. Regulation of Src Activity by Receptor Tyrosine Kinases (RTKs)

Src can acts as an upstream or downstream modulator of several receptor molecules, as well as nonreceptor tyrosine kinases, which are responsible for the robustness and persistence of RTK signaling [65]. Src acts as a signal transducer from the cell surface receptors by sequential phosphorylation of tyrosine residues on substrates [66]. Src participates in the activation of various downstream signaling pathways through molecular interactions with growth factor receptors such as the epidermal growth factor receptor (EGFR) family, hepatocyte growth factor receptor (Met), integrin cell adhesion receptors, steroid hormone receptors, G protein-coupled receptors, focal adhesion kinase (FAK) and cytoskeleton components [65, 67]. Src can activate the phosphatidylinositol 3-kinase (PI3K)-Akt, growth factor receptor-bound protein 2 (Grb2)-Ras-Raf-mitogen-activated protein kinase (MAPK), Jak-signal transducers and activators of transcription (STAT) as well as FAK-paxillin-p130-Crk-associated substrate (Cas) cascades that are most crucial for cell cycle progression, survival, and proliferation [68–72]. Aberrant expression and activation of Src occurs in several tumor types and has been correlated with poor clinical outcome, which has stimulated interest in using Src kinase inhibitors as therapeutic cancer agents, some of which have entered the clinical trial stage [73, 74].

A variety of Src-binding proteins have been detected that compete for binding to the protein's SH domains and disturb the intramolecular interactions that allow the activation of Src kinase. v-Src cellular counterpart (c-Src) forms activated dimerized receptors via its SH2 domain binding to specific phosphotyrosine residues in the platelet-derived growth factor receptor (PDGFR) juxtamembrane region [75]. Other reports have suggested that activated PDGFR can phosphorylate tyrosine residues in the SH2/SH3 domain of Src and subsequently activate Src [76–79]. FAK is another kinase molecule able to bind to the Src-SH2 domain and activate the kinase activity [80–82]. Additional examples of regulators are FAK binding partners p130Cas [83, 84] and PTP*α* [85]. Recently, p130Cas, a protein that is thought to function as a docking protein because of its large number of binding motifs, has been demonstrated to bind to Src-SH2 and SH3 domains, resulting in Src activation [84]. Nef [86] and Sin [87] are examples of proteins that can bind to SH3 domains and activate the Src-family members Hck and Src, respectively. 

There is also evidence to suggest that Src cooperates with EGFR in growth signaling [88, 89]. Src promotes EGF-induced anchorage-independent growth and tumorigenesis in nude mice. Cooperation between these two proteins depends on Src catalytic activity [90, 91]. EGFR leads to transient activation of Src kinase activity in glioma cells. Activation of Src leads to phosphorylation of Tyr845 on EGFR which is not an autophosphorylation site [92]. In an independent study on glioblastoma patients, Lu have shown that Src and Fyn act as effectors of oncogenic EGFR signaling and enhance invasion and tumor cell survival *in vivo*. Selective inhibition of Src and Fyn limited EGFR-dependent tumor cell motility. Src inhibition combined with an anti-EGFR monoclonal antibody further inhibited tumor growth and increased survival in an orthotopic glioblastoma mouser model [93]. Src is responsible for activation of STAT transcription factors after activation of ErbB1 by EGF, suggesting that EGF-induced mitogenesis might be mediated by the Src-STAT pathway which is independent of Jak [94]. Recently, we have shown that Src and c-Met interact differently in head and neck cancer cells that are sensitive or resistant to Src inhibition. Interestingly, however, in both cases c-Met acts as a direct Src substrate in an *in vitro* immunocomplex kinase assay system, which suggests that Src-dependent cell survival is also regulated by c-Met receptor activation, at least in head and neck cancer cells [95]. 

 Another tier of Src regulation by RTKs was demonstrated by Jiang et al. who showed that EGFR, PDGFR, and fibroblast growth factor receptor (FGFR) phosphorylate Cbp upon ligand stimulation [96]. The EGFR mediated Cbp phosphorylation occurs via Src. Overexpression of Cbp blocks EGFR-mediated Src activation, signaling, and cell transformation, whereas loss of Cbp function has the opposite effect. Thus, Cbp may regulate the synergistic interactions between Src and EGFR in breast cancer.

## 6. Focal Adhesion

In a manner similar to many other signaling molecules, Src exerts its biologic action not only through its enzymatic activity and multidomain structure but also through its ability to interact with other signaling molecules in different cellular compartments [97]. Due to its N-terminal fatty acid moiety, Src associates with the plasma membrane as well as the perinuclear and endosomal membranes. The inactive form of Src has juxtanuclear localization. Upon activation by phosphorylation, Src SH3 domain associates with actin filaments, which then drive the translocation of Src to cell-cell and cell-matrix adhesion sites, where Src can interact with plasma membrane-bound molecular partners to take part in two major transduction events. These are (i) signaling from receptor tyrosine kinases, which mainly affects cell growth, proliferation, and migration and (ii) signaling from adhesion receptors, including integrins and E-cadherin, which mainly regulate cytoskeletal functions [98]. Constraints on the SH2 and SH3 domains that are released when the molecule is activated and are also likely to influence intracellular signaling by allowing the recruitment of high-affinity binding partners to specific intracellular sites. By this means, conformational activation of Src induces formation of SH2- and SH3-dependent multiprotein complexes at the cell periphery.

The primary role of tyrosine phosphorylation is to generate docking sites for proteins containing SH2 or phosphotyrosine binding (PTB) domains, thereby promoting protein-protein interaction and the formation of the macromolecular complexes responsible for signal transduction [99]. Many prominent Src substrates are found in focal adhesion junctions and include FAK, Cas, and tensins. Focal adhesion junctions are the sites of integrin-dependent substrate adhesion.

Tensins are the members of focal adhesion proteins that can serve as Src substrates. There are four members of the tensin family in mammals [100]. Tensins 1–3 contain three distinct regions: the N-terminal domain, which binds to F-actin and targets molecules for focal adhesion, a nonconserved central region, and the C-terminal SH2- PTB domain. The SH2 domains of Tensin-1 are required for promigratory functions [101], and the SH2 domains of Tensin 2 and 3 are responsible for binding with proangiogenic tyrosine (Tyr)-phosphorylated Cas and FAK. Qian et al. showed for the first time that the knockdown of Tensin-3 inhibited Src mediated cell transformation as well as cell migration and the growth of cancer cell lines [102].

Previously, Davis et al. showed that Tensin-1 is Tyr phosphorylated in Src-transformed chicken embryo fibroblasts [103]. Qian et al. observed that in a panel of human cancer cell lines, the level of phospho-Tensin-3 correlated roughly with both malignancy and with the levels of Src kinase activity [104]. Furthermore, the level of phospho-Tensin-3 was strongly reduced by specific inhibition of Src. Tensin-3 was also phosphorylated at Tyr in a mouse mammary tumor virus-(MMTV-)polyoma middle T (PyMT) murine model, in which endogenous Src was activated. This phosphorylation was reversed by Src inhibitor PP2. In addition, recombinant Src was also able to phosphorylate Tensin-3 *in vitro * [102]. They also have shown that the Tyr residue of SH2 domain of Tensin-3 at positions 1173/1206 and 1256 was phosphorylated by Src in a range of different types of cancers. Interestingly, Src inhibitors not only decreased the phosphorylation of Cas and the RNA-binding protein Sam68, but also decreased its interactions with Tensin-3.

## 7. Src Localization

Studies on the subcellular localization of Src reveal that it has been associated with the plasma, perinuclear, and endosomal membranes [105–109]. Although much evidence has been acquired regarding the role of Src at the plasma membrane and its interaction with growth factor receptors and integrin-nucleated focal adhesion complexes for regulating cell growth and proliferation [8, 66, 97], the functional significance of Src at other subcellular locations, such as cytoplasmic granules and perinuclear membranes, has not been as well characterized. The punctate staining pattern of Src in fibroblasts may represent the protein's association with membrane vesicles. Furthermore, analysis of Src function in Src-overexpressing fibroblasts indicates a possible association between Src with endosomal membranes [110]. Analysis of indirect immunofluorescence by three-dimensional optical sectioning microscopy revealed Src to be associated primarily with membranes at the microtubule organizing center, which represent a late stage in the endocytic pathway [109]. Moreover, Src is also associated with a number of microtubule-related structures including microtubule bundles at point of cell-cell contact and a region associated with the spindle pole during mitosis that regulates the transport or function of specialized secretory vesicles [109]. 

These data contrast with and extend previous reports of Src localization at the plasma membrane. One explanation for this discrepancy was that the biochemical fractionation techniques used in some prior studies did not differentiate between the plasma and endosomal membranes, which have similar densities and are thus likely to cofractionate [109]. The presence of Src in secretory organelles of chromaffin cells and platelets [111, 112], its association with endosomally derived synaptic vesicles in differentiated PC-12 cells [113], and the development of osteopetrosis in mice that are null for Src [114] further suggest a possible role for Src in protein-trafficking events.

### 7.1. Perinuclear and Nuclear Signaling

Src exhibits a predominantly perinuclear pattern of expression in malignant cells in contrast to a more evenly cytoplasmic distribution in normal breast epithelial cells [115]. The localization of Src to perinuclear membranes, endosomes and possibly even the nucleus suggests that Src is involved in nuclear-signal transduction events. The tyrosine kinase activity of Src is increased in mitotic cells arrested with nocodazole [116]. There is growing evidence that Src may play a role in cell cycle regulation especially at the G1/S transition [117, 118]. A 68 kDa phosphorylated protein (Src associated in mitosis, SAM68) is associated with Src in Src-activated mouse fibroblasts. An identical 70 kDa protein was identified as a tyrosine phosphorylated protein that was capable of binding to Lck and regulating T-cell activation. It has been postulated that Src regulates general splicing and mRNA transport via its effects on the expression at the posttranscriptional level of Sam68 [119]. Comparison of several modes of Src activation demonstrates that Src could either slow down the splicing rate or allow the export of partially spliced transcript [119]. Overexpression of Fyn in HEK293 cells interferes with the association of Sam68 with the splicing factor YT521-B and demonstrates Fyn's role in mRNA splicing [120]. Gondran and Dautry further strengthen the importance of Src in mRNA splicing and transport by inducing mutations at the SH2 and SH3 domains in Src [121]. There is evidence that Src can interact with different SH2 and SH3 domains containing signaling molecules such as PLCg-1, Grb2, NCK, Jak3, SHP1, Cbl, Grap (Grb2 like protein), p21 GTPase, p85 subunit of PI3K, p47 and Tec kinase family [122–130]. ASAP1, an ADP-ribosylation factor, is associated with Src [131]. ASAP1 is found primarily in the cytoplasm in a perinuclear, reticulate network. The association of Src with ASAP1, Arfs and PIP2 is thought to be important in coordinating membrane trafficking with actin cytoskeletal remodeling [131, 132]. Src associates with and phosphorylates various proteins responsible for vesicle transport at the perinuclear region; such as synapsin, dynamin, and so forth, [133–135]. Golgin67 has also been identified as a potential Src target, involved in vesicle docking and tethering [136]. Collectively this evidence suggests that Src might have a role in membrane trafficking events through transgolgi network [137].

## 8. Involvement of Src in Human Cancers

Src contribution to cell regulation and cancer development has been widely discussed in several review articles [74, 97, 138], so the discussion will be limited to a very short summary of a few relevant concepts and experimental findings.

There is a large body of evidence that has demonstrated that Src kinase activity and protein levels are elevated in several cancers, including those of the colon and breast. A correlation has often been observed between increases in Src kinase activity and the progression of malignancy [62, 64, 97, 115, 139–141]. Previously, we showed that Src promotes cancer cell survival in conjunction with STAT3 in head and neck squamous cell carcinoma (HNSCC) and non-small cell lung carcinoma (NSCLC) cells [142, 143].

Recently, Zhang et al. provided both clinical and experimental evidence that Src plays a critical role in the establishment of latent bone metastasis in breast cancer [144]. Using a bioinformatic approach that investigated the association between various signaling pathway-specific gene expression patterns and breast cancer, they identified a “Src activity gene expression signature” (Src responsive signature, SRS) that was highly associated with late onset of bone metastasis in breast cancer. To address the role of Src in the process of bone metastasis, they used two SRS-expressing breast cancer cell lines that possessed either aggressive or indolent metastatic bone tropism in a xenograft mouse model. In the cell line possessing aggressive metastatic bone tropism, stable knockdown of Src resulted in a significantly decreased rate of tumor outgrowth of bone lesions. In an indolent model of bone metastasis, knockdown of Src led to complete loss of bone metastatic activity, whereas the silencing of Src did not alter lung or lymph node metastatic activity, thus supporting a specific role for Src in bone metastasis. These prominent findings set the stage for the development of novel therapeutic strategies for eradicating breast cancer metastasis to bone.

In 2009, Yim et al. showed that the ectopic expression of Rak (also known as Frk) effectively suppressed breast cancer cell proliferation, invasion, and colony formation *in vitro* and tumor growth *in vivo* via its regulation of PTEN protein stability and function. Thus Rak may function as a tumor suppressor gene. Further understanding of its function may contribute to effective therapeutic approaches for both Rak- and PTEN-defective cancers [145]. 

 Using integrated genomic and phosphoproteomic analysis of mouse lung primary and metastatic tumors, Carretero et al. demonstrated that loss of tumor suppressor LKB1 led to the activation of Src and FAK in a Kras^G12D^/Lkb1 murine model of lung tumor [146]. Src and FAK activation result in focal adhesion disassembly and turnover through the downregulation of Ras homolog gene family, member A (RhoA), which results in an increase in cellular motility and migration in the process of metastasis. They also confirmed the involvement of Src in the regulation of metastasis in Kras^G12D^/Lkb1 lung tumors by inhibiting Src, with concomitant increase in the sensitivity of tumor towards PI3K-MEK inhibition.

## 9. Clinical Trials of Src Inhibitors

A large body of evidence, including that discussed above, has identified Src as a key molecule in tumor progression that can provide oncogenic signals for cell survival, EMT, mitogenesis, and invasion and angiogenesis and metastasis [74, 147]. Due to the positive correlation between the development of cancer and the upregulation of Src activity, Src is emerging as a promising target for anticancer therapy [148, 149]. Src inhibition also results in a reduction of cancer progression in several cancer types [150–152], thus suggesting a potential clinical usefulness to inhibiting Src. There are several small molecule inhibitors for Src kinase that are undergoing clinical trials after promishing preclinical studies, such as the ATP-binding competitive inhibitors dasatinib (BMS-354825, Sprycel), bosutinib (SKI-606), saracatinib (AZD530), ponatinib (AP24534), bafetinib (INNO-406), and the substrate binding-site inhibitor Kxo-I (KX2-391) [153–157]. Preliminary data suggest that the agents are well tolerated at doses that achieve clinically meaningful plasma drug concentrations. Recent clinical studies with Src inhibitors as single agents or in combination are shown in Table 1.

Dasatinib suppressed invasion and induced cell cycle arrest in HNSCC cells *in vitro* [158], affected the mechanisms of prostate tumor progression [159], and greatly inhibited the development of liver metastasis in an orthotopic murine model of pancreatic carcinoma. Studies of dasatinib in prostate [160] and colon cancer cell lines [161] showed inhibition of cellular adhesion, migration, and invasion. Breast cancer cell lines belonging to the basal/“triple-negative” subtype were particularly sensitive to dasatinib. Breast cancers within this subgroup express basal cell cytokeratins (CK5 and CK17), with ER, PR and Her2 negative phenotype [162, 163], and are well known for poor prognosis [164]. Interestingly, in EGFR-overexpressing breast cancer cell lines, dasatinib inhibited cell growth, invasion, and angiogenesis, and stimulated apoptosis by activating caspase 8 and 9 [165].

Bosutinib showed activity against colon cancer in a murine model and was well tolerated. In cellular assays, bosutinib treatment resulted in a dose-dependent reduction in proliferation, invasion, and migration of breast cancer cells [166, 167]. Furthermore, in a murine model of breast carcinoma, bosutinib inhibited tumor growth and significantly reduced the number of liver, spleen, and lung metastases. Clinical trials with bosutinib for breast cancer, other solid tumors, and leukemia are ongoing [168]. 

Saracatinib (formerly AZD0530; AstraZeneca) is another ATP-competitive inhibitor of SFKs, with activity against ABL and activated mutant forms of EGFR (L858R and L861Q) [169]. In a panel of 13 human cancer cell lines treated with saracatinib, there was growth inhibition in four different cell lines (derived from colon, prostate, and lung tumors) and inhibitory effects on migration and invasion [170, 171].

In a recent phase II trial with dasatinib as a first line of treatment for metastatic NSCLC several patients had prolonged stable disease and one patient had a near complete response that persisted 2 years after the start of therapy, suggesting that there was a subset of patients with NSCLC who benefited from Src inhibition [172]. Another independent phase I/II study in NSCLC using the combination of Src and EGFR inhibitors also demonstrated clinical responses [173, 174]. These observations further validate the preclinical findings that suggest there is cooperation between EGFR kinase activity and Src in NSCLC [158, 175–177]. 

In a phase II trial in 2008, Yu et al. demonstrated that dasatinib improves the overall survival in castration resistant prostate cancer [178]. Based on promising results from phase I/II clinical trials of combination treatment with dasatinib and docetaxel in prostate cancer patients, this combination is now being tested in a phase III clinical trials [118, 119].

 M475271 is an oral inhibitor of Src and vascular endothelial growth factor receptor (VEGFR) that has shown preclinical activity in lung adenocarcinoma cell lines [179]. Another SFK inhibitor, KX2-391, targets the peptide substrate-binding site rather than the ATP-binding site. Based on the promising results from phase I study, a phase II study has been initiated with Castration-Resistant Prostate Cancer Bone-Metastatic patients [180], (http://www.clinicaltrial.gov/). All these therapeutic agents appear to be well tolerated and we eagerly await their detailed clinical results.

## 10. Conclusions

Our understanding of Src structure and function, regulation, and localization has increased dramatically since its discovery. One-hundred years after the original description of Src, this protein continues to attract keen interest because of its multiplicity of actions in the molecular signaling pathways underlying developmental as well as oncogenic events. Many studies have addressed the molecular mechanisms of Src regulation in cells and tumor tissues. In order to clarify and fully elucidate the normal physiologic function of Src and other SFKs and to fully comprehend Src signaling networks in various cancers, Src interactions with specific targets or binding partners in different subcellular localization studies should be characterized in as much detail as possible. Special focus should be placed on the role of Src in bone metastasis because of the protein's role in osteoclast and osteoblast function. Moreover, preclinical reports of combination treatments involving chemotherapy, radiation therapy, and targeted therapies with a Src inhibitor warrant further investigation [181, 182].

## Figures and Tables

**Table 1:** Src inhibitors with other agents in clinical trials.

Drug	Phase	Tumor type	Combination agent
Dasatinib	II	Advanced-NSCLC/Colorectal/Pancreatic/HNSCC/Breast/SCLC/Melanoma	—
	II	Resectable NSCLC/HNSCC	Erlotinib
	I-II	Advanced NSCLC	Erlotinib
	I	Breast	Capecitabine
	I	Breast	Paclitaxel
	I-II	Prostate/castration resistant prostate cancer	Docetaxel
	I	Colon	FOLFOX6/Cetuximab

Saracatinib	II	Prostate/Pancreatic/Osteosarcoma/Soft tissue sarcoma/Melanoma/Gastration-resistant prostate cancer/Thymoma/Colorectal/HNSCC	—
	II	Advanced NSCLC/SCLC	Carboplatin/Paclitaxel
	I	Advanced solid tumor	Cediranib
	I-II	Pancreatic	Gemcitabine
	II	Ovarian	Carboplatin
	II	Prostate/Breast with bone metastasis	Zoledronic acid

Bosutinib	II	Breast	—
	II	Breast	Exemestane
	II	Breast	Letrozole/Capecitabine
	I-II	Advanced solid tumor	Capecitabine

XL228	I	Advanced solid tumor	—

KX2-391	I	Advanced solid tumor/Lymphoma	—

AZM475271	I-II	Pancreatic	—

XL999	I	Advanced solid tumor	—
